# Psychosocial Intervention for Youth With High Externalizing Behaviors and Aggression Is Associated With Improvement in Impulsivity and Brain Gray Matter Volume Changes

**DOI:** 10.3389/fpsyt.2021.788240

**Published:** 2022-01-06

**Authors:** Nathan J. Kolla, Areti Smaragdi, George Gainham, Karolina H. Karas, Colin Hawco, Justin Haas, Tracey A. Skilling, Margaret Walsh, Leena Augimeri

**Affiliations:** ^1^Centre for Addiction and Mental Health (CAMH), Toronto, ON, Canada; ^2^Violence Prevention Neurobiological Research Unit, Toronto, ON, Canada; ^3^Department of Psychiatry, University of Toronto, Toronto, ON, Canada; ^4^Waypoint Centre for Mental Health Care, Penetanguishene, ON, Canada; ^5^Waypoint/University of Toronto Chair in Forensic Mental Health Science, Penetanguishene, ON, Canada; ^6^Stop, Now and Plan, Southampton, United Kingdom; ^7^Western University, London, ON, Canada; ^8^SickKids Hospital, Toronto, ON, Canada; ^9^University of Toronto, Toronto, ON, Canada; ^10^Child Development Institute, Toronto, ON, Canada

**Keywords:** externalizing behavior, aggression, impulsivity, cognitive behavioral therapy, structural magnetic resonance imaging

## Abstract

**Background:** Stop, Now And Plan (SNAP) is a cognitive behavioral-based psychosocial intervention that has a strong evidence base for treating youth with high aggression and externalizing behaviors, many of whom have disruptive behavior disorders. In a pre-post design, we tested whether SNAP could improve externalizing behaviors, assessed by the parent-rated Child Behavior Checklist (CBCL) and also improve behavioral measures of impulsivity in children with high aggression and impulsivity. We then investigated whether any improvement in externalizing behavior or impulsivity was associated with gray matter volume (GMV) changes assessed using structural magnetic resonance imaging (sMRI). We also recruited typically developing youth who were assessed twice without undergoing the SNAP intervention.

**Methods:** Ten children who were participating in SNAP treatment completed the entire study protocol. CBCL measures, behavioral measures of impulsivity, and sMRI scanning was conducted pre-SNAP and then 13 weeks later post-SNAP. Twelve healthy controls also completed the study; they were rated on the CBCL, performed the same behavioral measure of impulsivity, and underwent sMRI twice, separated by 13 weeks. They did not receive the SNAP intervention.

**Result:** At baseline, SNAP participants had higher CBCL scores and performed worse on the impulsivity task compared with the healthy controls. At the second visit, SNAP participants still had higher scores on the CBCL compared with normally-developing controls, but their performance on the impulsivity task had improved to the point where their results were indistinguishable from the healthy controls. Structural magnetic resonance imaging in the SNAP participants further revealed that improvements in impulsivity were associated with GMV changes in the frontotemporal region.

**Conclusion:** These results suggest that SNAP led to improvement in behavioral measures of impulsivity in a cohort of boys with high externalizing behavior. Improvement in impulsivity was also associated with increased GMV changes. The mechanism behind these brain changes is unknown but could relate to cognitive behavioral therapy and contingency management interventions, important components of SNAP, that target frontotemporal brain regions. Clinically, this study offers new evidence for the potential targeting of brain regions by non-invasive modalities, such as repetitive transcranial magnetic stimulation, to improve externalizing behavior and impulsivity.

## Introduction

Disruptive behavior disorders (DBDs) are characterized by symptoms of conduct-disordered behaviors and oppositionality that are among the most prevalent classes of problems affecting young children (1–4). Data suggest that one in 11 preschoolers meet diagnostic criteria for a DBD (1, 2, 5). Among the negative consequences of DBDs include risk for later life psychopathology, family dysfunction, and criminality (6–8).

Stop, Now and Plan (SNAP) is a cognitive behavioral-based psychosocial intervention for youth with high aggression and externalizing behaviors. SNAP has a rich evidence base for helping children improve their self-control and externalizing behaviors. For example, one investigation compared SNAP participants to wait-list controls (318 boys and girls) and reported significant improvements on the Child Behavior Checklist (CBCL) (9) and Social Skills Improvement Scale (10) among the SNAP youth (11). Wait-list controls were children who were on waitlists for SNAP treatment and who were not yet receiving SNAP. Other program evaluation studies compared 252 boys who were randomized to SNAP or standard community services as usual. At follow-up, CBCL scores on the aggression, conduct problems, and externalizing behavior were significantly decreased in the SNAP condition relative to treatment as usual. In fact, boys who displayed the most diverse behavioral difficulties evidenced the most improvement (12). SNAP model programs also show evidence for reducing offending (12), reducing conduct problems in boys with high levels of conduct problems (13), and decreasing financial costs of chronic delinquency to society (14). For example, a cost benefit analysis found that every $1 spent on SNAP yielded an estimated savings between $2.05 and $3.75 (14).

As mentioned, enhanced self-control is a key theoretical mechanism for change in the SNAP intervention (12). Self-control has been described as a distinctive individual concept that is reliably associated with individuals' capacity to override immediately rewarding behaviors and engage in continued, effortful, goal-oriented behavior (15–17). Impulsivity may represent an important contributory factor to self-control (18). In impulsive choice, self-control relates to the ability to delay gratification and select the larger, delayed reward. Self-control can also be expressed by choosing the smaller, certain reward versus the temptation of gambling and selecting the larger, uncertain reward (19). Interestingly, longitudinal analysis revealed that physiological self-regulation or control moderated the effect of trait impulsivity on externalizing behaviors (20) in a non-clinical sample of adolescents, while another study of a community sample concluded that the effects of stress on self-control were mediated by impulsivity (18).

To the best of our knowledge, only one study has investigated biomarkers of change among children participating in an evidence-based, multimodal treatment for high externalizing behavior. This investigation examined neurobiological changes associated with positive outcomes related to self-regulation in SNAP. In this study, neural markers of self-regulation using electroencephalography in SNAP participants aged 8–12 years old were measured (21). Compared with a non-clinical, age- and gender-matched control group, SNAP participants were found to have larger N2 magnitudes and smaller frontal P3 magnitudes. Interestingly, SNAP children who improved with treatment exhibited a significant decrease in the magnitude of the N2 versus children who did not improve. Among the children who improved, source analysis during the time of the N2 evidenced a reduction in activation of the ventral and dorsomedial prefrontal cortex, in addition to the anterior medial temporal lobe. Reductions in activation of these brain regions are consistent with improvement in self-control (22, 23).

Emboldened by these results, we originally planned a task-based functional magnetic resonance imaging (fMRI) experiment of inhibitory control in SNAP youth, while also collecting structural imaging data. We intended to study the relationship between pre-post fMRI data and any changes in clinical and behavioral measures of the SNAP participants. Unfortunately, participant motion in the MRI scanner was too severe to use the majority of the functional data. As a result, we analyzed the well-preserved structural imaging data to investigate whether other biomarkers could be plausibly linked to anticipated improvements in clinical and behavioral measures among the SNAP participants. Accordingly, we investigated whether SNAP treatment would lead to improvement in clinical and behavioral measures and whether any improvement would be associated with brain gray matter volume (GMV) changes using structural magnetic resonance imaging (sMRI).

## Methods

### Ethics

This study was approved by the Centre for Addiction and Mental Health (CAMH) Research Ethics Board in Toronto, Ontario, Canada. All participants provided informed written consent prior to enrollment. All parents of the children participants signed informed consents for the study, while all children who participated in the investigation signed age-appropriate informed assent forms. All methods were performed in accordance with the relevant guidelines and regulations.

### Clinical Trial

This study was registered as a Clinical Trial: ClinicalTrials.gov ID: NCT0299807. The protocol ID is 0702016.

### Study Intervention

SNAP is an evidence-based psychosocial intervention that is aimed at 6–11-year-olds who present with externalizing behaviors, many of whom have DBDs (11). The 13-week core cognitive-behavioral groups (SNAP Boys and SNAP Girls) component teaches physiological awareness of emotional responding that may promote aggressive behaviors, and it also helps to improve self-control and impart problem-solving skills to make more adaptive choices. Central to the program is working with affected children, their families, peers, and schools during critical developmental stages. Another core SNAP component includes a concurrent 13-week SNAP Caregiver Group that focuses on effective parent management strategies and also helping caregivers learn the SNAP strategy to support their child. Supplementary program components include individual child counseling and mentoring, family counseling, school advocacy, teacher consultation, and SNAP youth leadership. The focus of each SNAP component is to generalize SNAP strategy skills across a multitude of settings. The SNAP model adopts a holistic treatment approach emphasizing the importance of how self-control and self-regulation fit in the broader context of risk and prosocial factors.

As discussed above, SNAP was offered to children, which is based on a cognitive behavioral therapy (CBT) model and parent management training. The two core SNAP components, SNAP Boys/SNAP Girls Group and the SNAP Caregiver Group are delivered for 90 min concurrently each week over 13-weeks at community agencies. CBT promotes efficient regulation of emotion and impulses through strategies such as cognitive restructuring, problem-solving, role-playing, social and token reinforcements, and generalization activities (24). Parent management training imparts positive parenting practices, including skill encouragement, problem-solving, and monitoring in addition to substitution of coercive or lax discipline strategies with mild sanctions addressing misbehavior (25, 26).

### Participants

Thirteen SNAP participants were recruited and 12 sex-matched healthy control participants were recruited for the study. SNAP participants were recruited from the group of children who were commencing SNAP treatment at a community agency. The study was explained to SNAP children and their parents prior to the beginning of treatment for different SNAP treatment groups held during the lifespan of the study. Interested individuals were then encouraged to contact the Research Analyst leading the study. Healthy controls were recruited from the community in response to social media posts. All participants across both groups were male and right-handed. The only inclusion criteria for the SNAP children were that they were male and that they were participating in SNAP. Three SNAP participants were excluded from analysis as two individuals withdrew from the study (not SNAP treatment) before their post-SNAP second visit and another participant was unable to attend the post-SNAP second visit due to the COVID-19 pandemic. Thus, we had complete clinical, behavioral, and sMRI data for 10 SNAP participants and 12 healthy control participants.

### Study Variables

Demographic and clinical variables of interest included the following: age, ethnicity, handedness, past traumatic brain injury, and socioeconomic status (SES). SES was measured using the Nam-Powers-Boyd scale (27). Full-scale IQ was measured using the Wechsler Abbreviated Scale of Intelligence [WASI-II; (28)], which is a reliable and well-validated tool for the assessment of cognitive ability in individuals age six to 89 years. Independent samples *t*-tests were used to compare continuous variables, and chi-square tests were employed to compare categorical variables. Data were analyzed using SPSS 25 (IBM Corp., 2017). Significant values were defined by *p*-values < 0.05.

Psychiatric diagnoses were generated using the Kiddie Schedule for Affective Disorders and Schizophrenia for DSM-5 criteria [K-SADS-PL5; (29)], which is a semi-structured interview tool. All SNAP participants met criteria for a DBD, save one participant who was only diagnosed with attention deficit hyperactivity disorder (ADHD). Testing positive for any psychiatric disorder was an exclusion criterion for the healthy group participants. Diagnoses for the SNAP participants are presented in Table 1.

**Table 1:** Psychiatric diagnoses among the SNAP participants.

**SNAP participant**	**K-SADS-PL5 diagnoses**
SNAP 1	ADHD–predominately hyperactive-impulsive type and ODD
SNAP 2	CD
SNAP 3	ADHD–predominately inattentive type and ODD
SNAP 4	ODD
SNAP 5	ADHD–predominately inattentive type and ODD
SNAP 6	ADHD–predominately inattentive type
SNAP 7	ADHD–predominately hyperactive-impulsive type and ODD
SNAP 8	ODD
SNAP 9	ADHD–predominately inattentive type and ODD
SNAP 10	ADHD–predominately hyperactive-impulsive type and ODD

*ADHD, Attention Deficit Hyperactivity Disorder; ODD, Oppositional Defiant Disorder; CD, Conduct Disorder*.

### Outcome Measures

Because of the focus on the CBCL in prior SNAP studies, we designated parent-rated CBCL scores as a measure of psychosocial functioning that was obtained pre- and post-SNAP visits for the SNAP participants and pre- and post-visits, separated by 13 weeks, for the healthy controls for consistency/replication purposes. The CBCL is an extensively validated measure administered to a child's caregiver to assess various domains of psychopathology in youth. The measure consists of 112 items that the respondent can endorse as “not true,” “sometimes true,” or “very true.” The CBCL provides broadband (e.g., Internalizing and Externalizing) and narrowband (e.g., Anxious/Depressed, Withdrawn/Depressed, Aggressive, and Rule-Breaking) scales, with higher scores signaling the presence of impaired functioning on the characteristic of interest. Parents were directed to answer the CBCL questionnaire based on their child's presentation at the beginning of SNAP treatment and at the post-SNAP assessment period.

We also explored a laboratory measure of impulsivity as an outcome measure. The measure used was the computerized Two Choice Impulsivity Paradigm [TCIP; (30)]. The TCIP is a discrete-choice task used to assess preference for a smaller, immediate reward or a larger, delayed reward. Participants were instructed to earn as many points as possible, such that a proportional amount of points was rewarded according to the amount of time the participant chose to wait to make the selection. For example, if participants chose to wait for 5 s (e.g., the immediate choice), they received five points. Alternatively, participants were awarded 10 points for choosing to wait 10 s (e.g., the delayed choice). Participants who chose to wait less time earned less points and were deemed more impulsive compared with participants who chose to wait longer and earn more points. Thirty trials of the TCIP was employed as the threshold. Variables of interest for the TCIP included the number of immediate choices, the proportion of immediate choices, and the highest maximum consecutive number of immediate choices. We operationalized higher values for these variables as indicative of greater impulsivity.

### Statistics

In order to elucidate group × interaction effects for CBCL and impulsivity measures, we utilized a generalized estimating equation. All models were performed with an exchangeable working correlation matrix. Due to distributions being skewed or otherwise having a non-normal distribution, gamma or negative binomial distribution models were used, both with a log link function, depending if data were continuous or count variables, respectively. However, when the data had a symmetrical appearing distribution, a normal model with identity link was used. There were two levels for each variable: SNAP participant and healthy control and time 1 and time 2 (initiation of SNAP and post-SNAP for the SNAP participants and time 1 and time 2 separated by 13 weeks for the healthy controls). A *p*-value < 0.05 indicated significance.

### MRI

All participants underwent two MRI scanning sessions. For the SNAP participants, they were scanned at the start of SNAP treatment (time 1) and post-SNAP (time 2). For the healthy controls, they were scanned 13 weeks apart. All MRI scans were conducted using a 3T GE MR750 scanner (MR750, GE Healthcare, Milwaukee, USA) located at CAMH, Toronto, ON. Structural images were captured with a sagittal three-dimensional magnetization, which prepared three-dimensional, inversion recovery prepped, fast spoiled gradient echo (3D IR FSPGR) sequences (TR = 6.7 ms; TE = 3.0 ms; TI = 650 ms; frequency FoV = 230 × 230 mm; slice thickness = 0.9 mm; flip angle = 8°; matrix = 256 × 256; voxel size = 0.9 × 0.9 × 0.9 mm^3^; acquisition time = 4:41 min). T1 images were visually inspected for abnormalities. Pre-processing and voxel-based morphometry (VBM) analyses were completed in Statistical Parametric Mapping 12 [SPM12; (31)] running with MATLAB R2016a (MATLAB, 2016). Structural images were automatically segmented into three tissue classes: gray matter (GM), white matter (WM), and cerebrospinal fluid (CSF) (32). Subsequent inter-subject alignment and spatial normalization was performed using DARTEL (33–35) with a Gaussian kernel of 10 mm (FWHM).

GM volume differences in the SNAP group between time 1 and time 2 were analyzed with a multiple regression factorial design in the VBM option of SPM12. Because the current study aimed to investigate the SNAP program effect on CBCL measures and/or impulsivity measures, regression analyses were performed for those variables where SNAP participants were significantly improved at time 2 vs. time 1, relative to healthy participants. Change in brain volumes following SNAP treatment were calculated by subtracting the VBM GM volumes at time 1 from time 2, using fslmaths [https://fsl.fmrib.ox.ac.uk/fsl/fslwiki/Fslutils; (36)]. For each variable, a change score was calculated by subtracting the impulsivity baseline score from the post-treatment score. Age at second scan and total intracranial volume (TIV) were included as covariates in the analyses. TIV was calculated by summating participant-level GM, WM, and CSF volumes and averaging across both MRI scans. Tissue volume calculations were performed using the “Tissue Volume” utility in SPM12, which requires segmentation files outputted from the segmentation step during pre-processing. Voxel extent threshold was set at *p* < 0.001 and clusters were considered significant at *p* < 0.05 FDR, corrected for cluster size. Further information and support for the post-processing analysis adopted by this investigation can be found in the following reference: (37) and websites: (https://www.jiscmail.ac.uk/cgi-bin/wa-jisc.exe?A2=ind1705&L=SPM&P=R26026; https://www.jiscmail.ac.uk/cgi-bin/wa-jisc.exe?A2=ind1310&L=SPM&P=R1986).

## Results

Regarding demographic variables, the SNAP participants were older than the healthy participants (10.6 ± 1.3 vs. 8.9 ± 1.8 years). Therefore, age was used as a covariate for all subsequent analyses. There was no significant difference between groups for any of the other demographic variables. Demographic data are presented in Table 2.

**Table 2:** Clinical and demographic variables of SNAP and control youth at time 1.

**Clinical and demographic variables (time 1)**	**Healthy • (*n* = 12)**	**SNAP • (*n* = 10)**	**Statistics**	***p*-value**
Age (years)^a^	8.9 ± 1.8	10.6 ± 1.3	*t* = 2.5	0.022
Sex (M/F)	12/0	10/0	/	/
Handedness (R/L)	12/0	10/0	/	/
Race^b^	/	/	χ2 = 7.1	0.13
Caucasian (#)	5	5	/	/
Black (#)	2	0	/	/
Asian (#)	3	0	/	/
Hispanic (#)	0	2	/	/
Mixed (#)	2	3	/	/
SES^a^^π^				
Father's Boyd-NP Score	68.2 ± 22.8	60.3 ± 31.0	−0.63	0.54
Mother's Boyd-NP Score	55.6 ± 34.6	56.4 ± 29.9	0.053	0.96
Summated Boyd-NP Score	121.4 ± 47.4	112.7 ± 62.1	−0.33	0.75
WASI-II–Full Scale IQ^a^^§^	109.0 ± 10.7	99.8 ± 14.9	−1.5	0.15
Presence of Previous Traumatic Brain Injury (#)	1	1	/	/
CBCL^a^^Ω^				
Subscale “Activities”	10.8 ± 3.3	12.3 ± 0.45	1	0.33
Subscale “Social”	7.4 ± 2.5	7.2 ± 2.8	−0.12	0.91
Subscale “School”	4.8 ± 0.81	2.9 ± 1.3	−4.1	0.001
Total Competence Score	23.0 ± 5.9	22.3 ± 1.9	−0.28	0.78
Item 1 “Anxious/Depressed”	2.0 ± 2.6	8.9 ± 5.1	3.9	0.001
Item 2 “Withdrawn/Depressed”	1.3 ± 1.6	2.6 ± 2.4	1.2	0.17
Item 3 “Somatic Complaints”	1.0 ± 1.7	3.3 ± 2.5	2.5	0.021
Item 4 “Social Problems”	1.5 ± 1.6	7.3 ± 3.7	4.7	<0.001
Item 5 “Thought Problems”	1.7 ± 1.6	4.8 ± 2.3	3.6	0.002
Item 6 “Attention Problems”	2.3 ± 2.6	11.7 ± 2.8	7.8	<0.001
Item 7 “Rule-Breaking Behavior”	1.1 ± 1.6	6.2 ± 3.7	4.1	0.001
Item 8 “Aggressive Behavior”	3.3 ± 4.2	16.9 ± 7.7	5.1	<0.001
Item “Other Problems”	3.09 ± 1.6	9.1 ± 6.0	3.2	0.005
Internalizing Score	4.3 ± 4.7	14.8 ± 10.7	3.4	0.003
Externalizing Score	4.4 ± 5.5	23.1 ± 10.3	5.2	<0.001
Total Score	16.9 ± 13.4	70.4 ± 24.7	6.2	<0.001
Two Choice Impulsivity Paradigm^a^				
Number of Immediate Time Choices	5.0 ± 4.5	12.8 ± 7.0	−4.4	<0.001
Proportion of Immediate Choices	0.17 ± 0.15	0.43 ± 0.23	−5	<0.001
Highest Number of Consecutive Immediate Choices	1.2 ± 0.41	4.0 ± 3.4	−4.1	<0.001

a *independent samples t-test;*

b *chi-square test;*

π *socioeconomic status;*

§ *Wechsler Abbreviated Scale of Intelligence–Second Edition;*

Ω *Child Behavior Checklist–Parent Version*.

**Figure 1:**
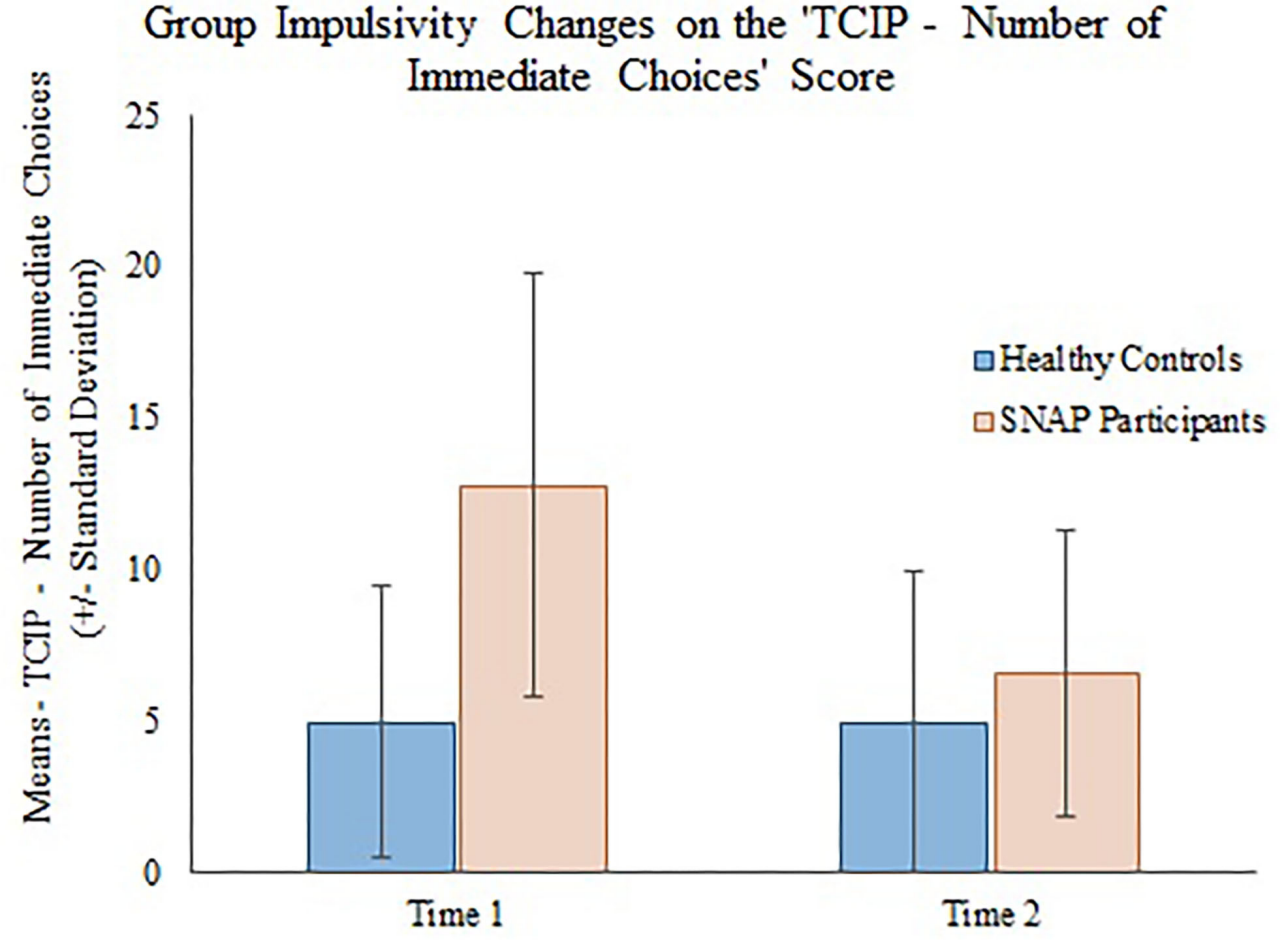
Group impulsivity changes on the Two Choice Impulsivity Paradigm–Number of Immediate Choices score.

**Figure 2:**
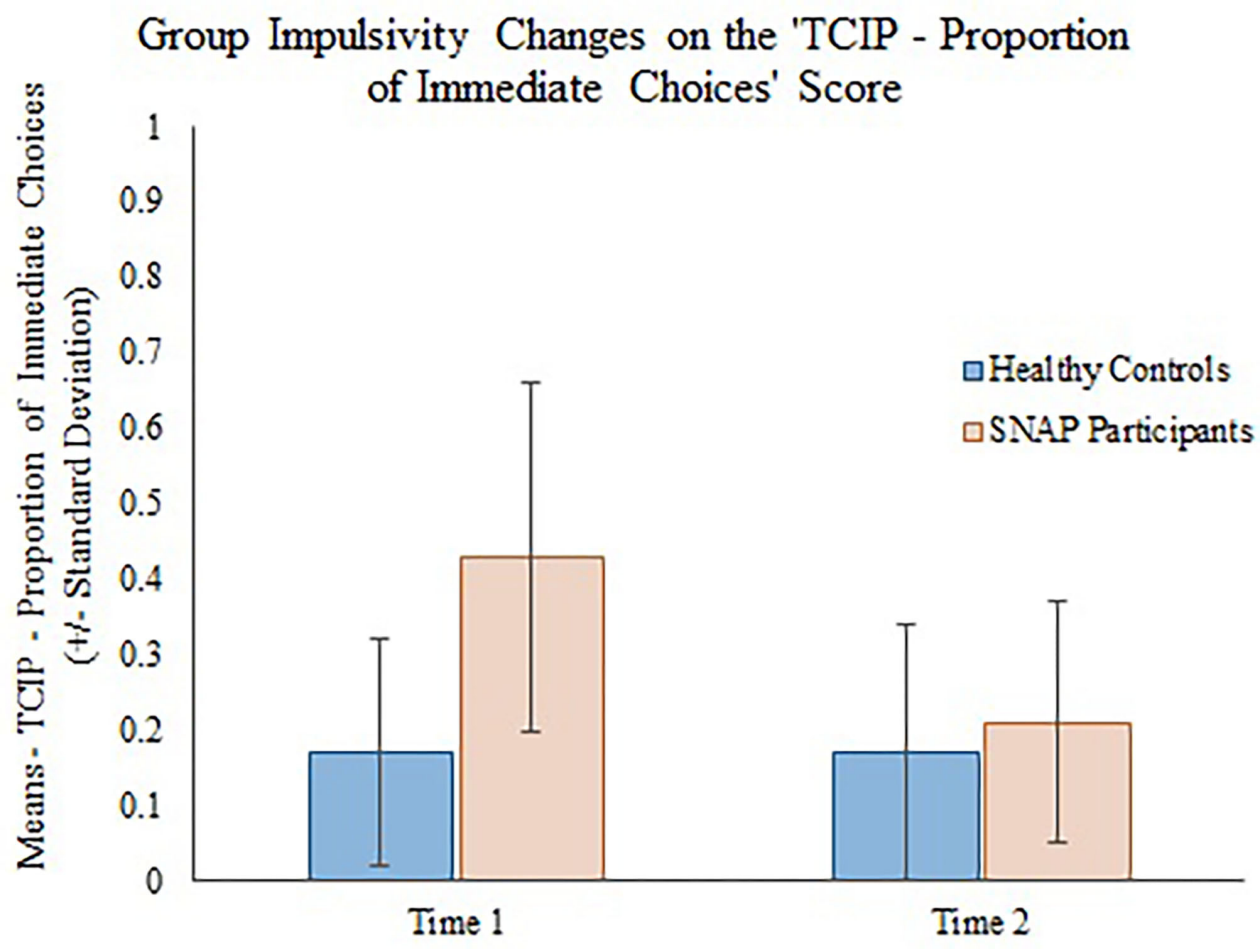
Group impulsivity changes on the Two Choice Impulsivity Paradigm–Proportion of Immediate Choices score.

**Figure 3:**
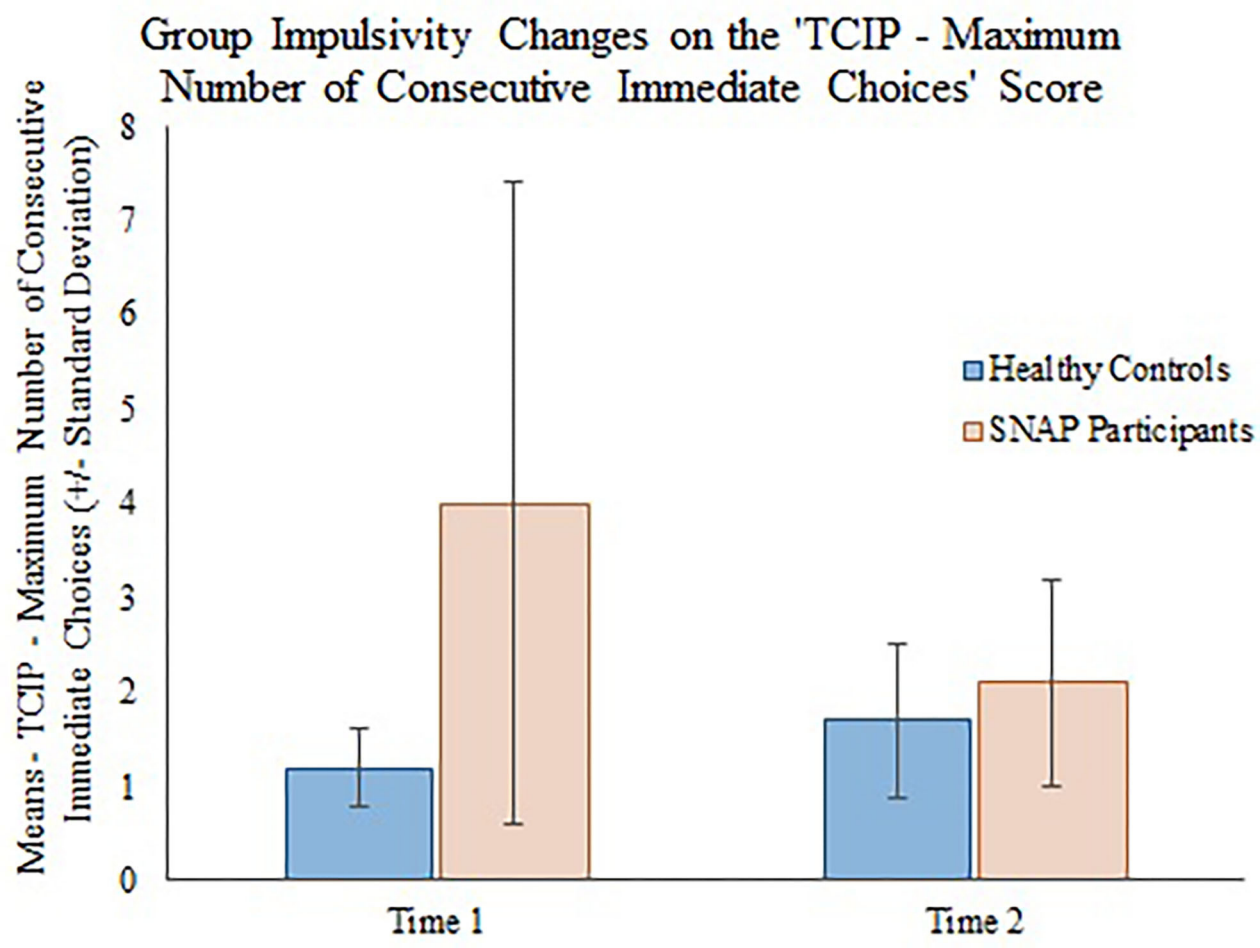
Group impulsivity changes on the Two Choice Impulsivity Paradigm–Maximum Number of Consecutive Immediate Choices score.

The generalizing estimating equations analysis yielded non-significant results for all CBCL variables. However, significant group × time effects were detected for all three measures of impulsivity. Results revealed that the TCIP number of immediate choices, proportion of immediate choices, and maximum number of consecutive immediate choices were all significantly lower in the SNAP group at time 2. Results similarly revealed that healthy controls had no significant change in their impulsivity scores from time 1 to time 2 (see Table 3, Figures 1–3).

**Table 3:** Group × time interaction effects for clinical variables measured with a generalized estimating equations model.

	**Wald χ^2^**	***p*-value**
CBCL^Ω^		
Subscale “activities”	0.038	0.85
Subscale “social”	0.44	0.51
Subscale “school”	0.31	0.58
Total competence score	0.037	0.85
Item 1 “anxious/depressed”	0.41	0.52
Item 2 “withdrawn/depressed”	1.2	0.28
Item 3 “somatic complaints”	0.81	0.37
Item 4 “social problems”	2	0.15
Item 5 “thought problems”	0.75	0.39
Item 6 “attention problems”	0.35	0.55
Item 7 “rule-breaking behavior”	1	0.32
Item 8 “aggressive behavior”	1.9	0.17
Item “other problems”	2.6	0.11
Internalizing score	0.1	0.75
Externalizing score	1.4	0.24
Total score	0.13	0.72
Two choice impulsivity paradigm		
Number of immediate time choices	7	0.008
Proportion of immediate choices	7.6	0.006
Highest number of consecutive immediate choices	6.7	0.01

Ω *Child Behavior Checklist–Parent Version*.

**Figure 4:**
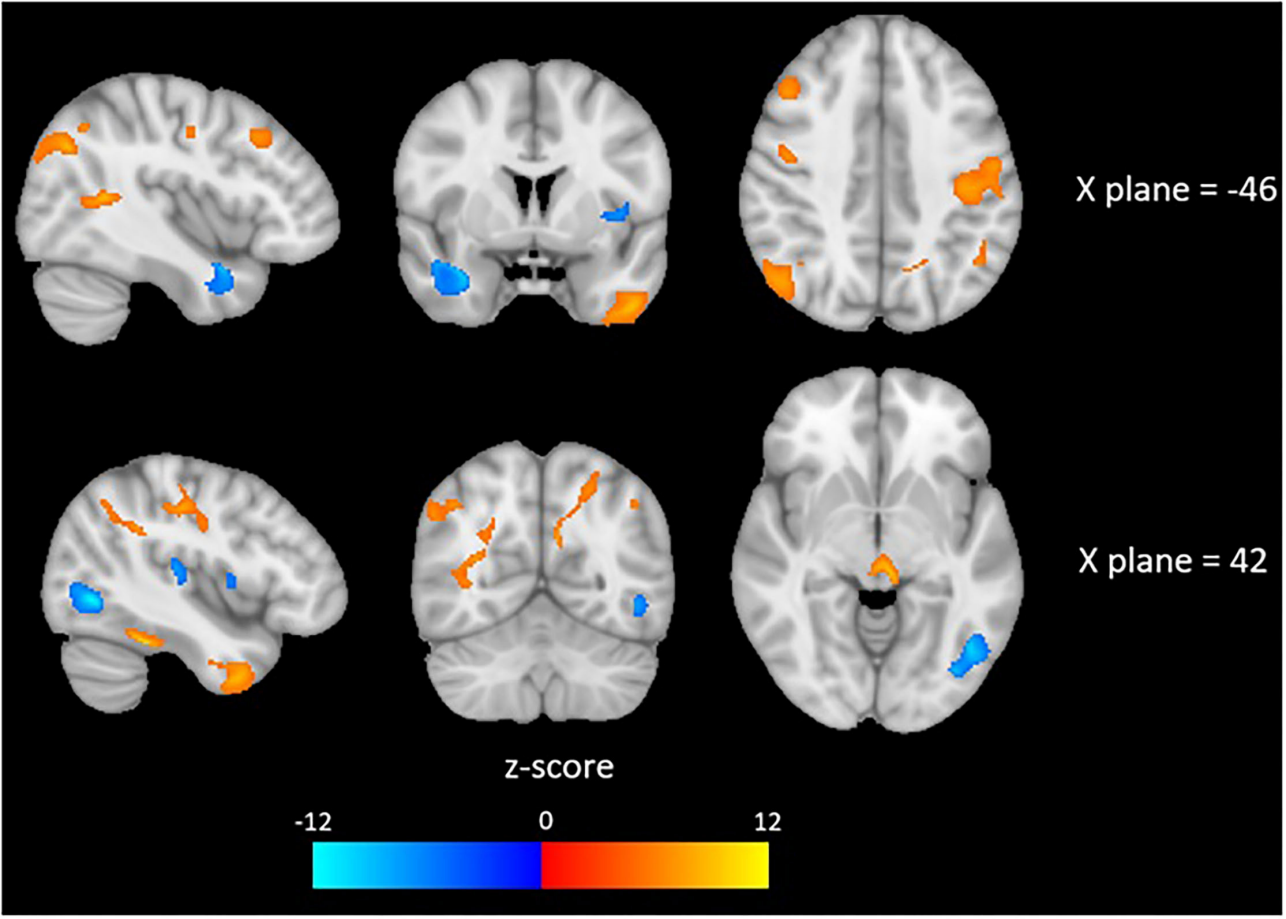
Red colors indicate a positive association and blue colors indicate a negative association between depicted brain areas and impulsivity, as measured by the Two Choice Impulsivity Paradigm–Maximum Number of Consecutive Immediate Choices score.

Brain imaging analysis demonstrated significant correlations between change in brain volume following SNAP treatment and the change score (e.g., improvement) for the impulsivity measure maximum number of consecutive immediate choices. Specifically, positive associations were detected in largely frontotemporal regions in the following clusters (*p* < 0.05, FDR-corrected at peak level): left middle temporal gyrus, right inferior temporal gyrus, right postcentral gyrus, right superior frontal gyrus, right temporal pole, left postcentral gyrus, and the left lateral occipital cortex. These results suggest that reduced impulsivity was associated with increases in the volumes of the aforementioned brain regions at time 2 in the SNAP sample. Negative correlations were detected in the following clusters (*p* < 0.05, FDR-corrected at peak level): left temporal pole, right lateral occipital cortex, and right intracalcarine cortex. These results suggest that improvements in impulsivity were associated with decreases in the volumes of the aforementioned brain regions at time 2 in the SNAP sample. The two other measures of impulsivity (e.g., number and proportion of immediate choices) were not associated with any GMV changes (see Figure 4 and Table 4).

**Table 4:** Positive and negative associations between gray matter volume and positive impulsivity change score.

**Condition**		**Brain region**	**FDRc**	**Number of voxels**	**MNI (mm)**		
					**x**	**y**	**z**
TCIP–maximum number of consecutive immediate choices	Positive association with improvement in impulsivity	Left middle temporal gyrus, temporooccipital part	<0.001	792	−39	−50	10
		Right inferior temporal gyrus, temporooccipital part	<0.001	2,438	46	−40	−21
		Right postcentral gyrus	<0.001	1,126	38	−26	50
		Right superior frontal gyrus	<0.001	920	12	−8	70
		Right temporal pole	<0.001	1,014	45	4	−40
		Left postcentral gyrus	<0.001	795	−58	−14	26
		Left lateral occipital cortex, superior division	<0.001	882	−42	−68	34
	Negative association with improvement in impulsivity	Right lateral occipital cortex, inferior division	0.001	637	44	−66	−3
		Left temporal pole	0.003	532	−34	3	−26
		Right intracalcarine cortex	0.001	785	2	−87	2

*FDRc, False Discovery Rate, corrected; MNI, Montreal Neurological Imaging Template; TCIP, Two-Choice Impulsivity Paradigm*.

## Discussion

This exploratory clinical trial aimed to test whether SNAP treatment would improve parent-rated CBCL scores and behavioral measures of a delay discounting impulsivity task. We then investigated whether any clinical improvement in these variables would be associated with GMV changes assessed using sMRI. We found that SNAP was associated with improvement on all behavioral measures of impulsivity but was not associated with change in CBCL scores. We also determined that improvement in one behavioral measure of impulsivity was associated with GMV changes in the SNAP sample post-treatment. From the outset, we emphasize that all results should be interpreted with caution given the relatively small sample size and exploratory nature of the analyses.

Our main finding was that all behavioral measures of a delay discounting task were significantly improved in the SNAP participants post-treatment. Prior to the SNAP treatment, the SNAP cohort performed worse on the task compared with the healthy controls, as evidenced by the selection of greater number and proportion of immediate choices and selection of a greater maximum number of consecutive immediate choices. However, after SNAP treatment, the SNAP participants' results had improved to the level where there was no distinguishable difference in performance between the SNAP participants and healthy controls at time 2. Because SNAP participants received interventions designed to improve self-control between time 1 and 2, whereas the healthy group did not receive an intervention, we attribute the improvement in the SNAP participants to the effects of SNAP treatment. Several components of the SNAP program could have contributed to positive change. Some, but not all investigations (38), demonstrate that CBT can reduce impulsivity and improve self-control in clinical populations (39). For example, a 12-week course of CBT was shown to reduce impulsivity, ascertained by improvement on Barratt Impulsiveness Scale 11 (40) scale scores, in a sample of depressed outpatients at the end of treatment and at follow-up 30 days later (41). In outpatient substance abusers (42), 12 weeks of group CBT treatment was associated with reductions in UPPS Impulsive Behavior Scale (43) scores. Contingency management (CM), which aims to influence decision-making processes by shifting preference for delayed versus immediate rewards (44), has also been shown to reduce delay discounting among substance users (45, 46). Given that SNAP is predicated on a CBT model that includes social and token reinforcement (11, 24), we tentatively suggest that CBT and CM interventions may have contributed to reductions in delay discounting among SNAP participants post-treatment.

It is also possible that the parent management training component of SNAP contributed to the improvement in impulsivity seen in the SNAP youth. There is evidence that parent skills training interventions can reduce impulsivity in youth with externalizing disorders. For example, one pre-post study of a structured parent skill training intervention found that parent-rated impulsivity of children with ADHD significantly improved after completion of the intervention (47). Therefore, modifications in the family environment may have also been responsible for the reduced impulsivity observed among the SNAP participants.

Contrary to other studies investigating the impact of SNAP on externalizing behaviors, we found that CBCL scores of SNAP participants were not significantly changed post-intervention compared with healthy controls. One possibility to explain this discrepancy is that we did not assess participants for changes in CBCL scores beyond time 2. Previous research has demonstrated that improvement on primary measures of antisocial behavior may be greatest at 15 months from baseline assessment (12). Thus, it is possible that had we assessed the SNAP participants beyond 13 weeks, we would have seen improvement on externalizing behaviors. Given that the CBCL is validated for answering questions about children's presentation during the past six months, it may not have been an ideal measure for assessing short term change. Anecdotally, it is often the case that CBCL scores do not decrease or, conversely, actually increase immediately following SNAP treatment, presumably because parent training during SNAP treatment makes parents more cognizant of their children's issues and behaviors and more likely to rate their children higher on the CBCL. However, it is also possible that our study was under-powered to detect changes in CBCL scores. Future investigations assessing neurobiological mechanisms of clinical improvement in SNAP participants should consider recruiting larger sample sizes.

We found that improvement on the delay discounting impulsivity task was associated with GMV changes in the SNAP participants. Most of the alterations involved increases in GMV in frontotemporal regions at time 2 that correlated with less delay discounting. The brain regions implicated in the current investigation are consistent with those identified in the SNAP EEG study that predicted positive change, namely the prefrontal cortex. Studies examining the neural networks underlying impulsivity consistently link it with network-level alterations in the prefrontal cortex and temporal cortex (48–51). For example, in healthy adults, lower trait impulsivity was correlated with greater cortical thickness in left middle frontal, orbital frontal, and superior frontal regions (52), whereas in a clinical population with high impulsivity, lower trait impulsivity was associated with greater left and right temporal cortex thickness (53). Delay-discounting tasks appear, in particular, to recruit frontotemporal and limbic neural systems (54, 55). According to one model of delay discounting, functioning of the lateral temporal lobe is germane to cognitive processes, such as theory of mind, and reflection on one's own mental state (56–59), while the medial temporal lobe is pertinent to simulation of potential future experiences and creation of mental images (59, 60). Thus, the cognitive functions of these regions can be understood in the context of mental tasks required of delay discounting, including imagining the future and contemplating one's own preferences (61). What is the mechanism behind changes in GMV volume following SNAP treatment? We can only speculate on this point, but one possibility is that CBT interventions promoted brain changes in the SNAP participants. For example, pre- and post-intervention studies in patients with chronic pain and chronic fatigue syndrome found that CBT treatment was associated with increases in prefrontal GMV (62, 63). Several reports have documented the up-regulation of neuroplasticity in rodents and mammals following enrichment of the environment (64–66). Accordingly, we cautiously propose that CBT interventions may have led to increased neurogenesis in frontotemporal regions among the SNAP cohort. This information is important clinically, because it pinpoints brain regions that may be amenable to biological treatment interventions, such as repetitive transcranial magnetic stimulation, that could help reduce impulsivity and externalizing behaviors.

Another potential explanation for the increased GMV following SNAP psychosocial treatment is that SNAP may have upregulated levels of brain derived neurotrophic factor (BDNF). BDNF is the best studied member of the growth factor neurotrophic family and is the most prevalent growth factor in the central nervous system (CNS) (67). BDNF plays a critical role in development and plasticity of the brain and is implicated in the pathology of many psychiatric disorders (68). Several studies have now reported that CBT and other behavioral therapies can increase peripheral levels of BDNF (69, 70). For example, one recent study found that CBT for chronic stress increased serum levels of BDNF, in addition to improving sleep, depressive symptoms, and emotional exhaustion (70). Some evidence suggests that serum levels of BDNF correlate with markers of neuronal integrity in the CNS (71). Therefore, we cautiously propose that increased GMV following SNAP treatment may have been associated with altered levels of BDNF that could promote neural plasticity. Future biochemical studies of BDNF and/or magnetic resonance spectroscopy studies of interventions for youth with high externalizing behaviors would be needed to test this hypothesis.

Several limitations of the current study must be noted. First, the sample size of the experimental group was relatively small. However, we were able to recruit a comparable control group. As previously noted, our smaller sample size may have rendered some analyses under-powered to detect significant change for some measures. In general, we found it challenging to recruit a sample of youth with high externalizing behavior who were willing to present twice for assessment of clinical variables and MRI scanning who were at the same time undergoing SNAP treatment. To promote future neurobiologically-informed research of aggression and externalizing behavior in youths, robust treatment programs, such as SNAP, may consider embedding scanning and other biological measures into their treatment protocol. Second, we did not consider any potential effects of psychotropic medication on the results obtained. Two SNAP participants were taking psychotropic medication throughout the duration of SNAP (one participant was taking a psychostimulant and another participant was taking a psychostimulant and an antidepressant). Given the small number of SNAP participants, it was not feasible to conduct subgroup analyses involving the medicated participants. Third, as we conducted group-level analyses, it was not possible to determine whether improvement on the impulsivity task was linked to GMV changes at the individual level.

In conclusion, these preliminary results demonstrated that SNAP treatment was associated with improvement on a delay discounting impulsivity task and GMV changes. Overall, there is very little research investigating biomarkers of change in children undergoing evidence-based treatment for externalizing and conduct-disordered behavior. This exploratory study adds to the literature base by using MRI to detect GMV changes following SNAP treatment. Although we caution the interpretation of our results given the smaller sample size, we believe that this work is still valuable as it provides a foundation for neurobiologically- informed research underlying improvement in impulsivity.

## Data Availability Statement

The raw data supporting the conclusions of this article will be made available by the authors, without undue reservation.

## Ethics Statement

This study involving human participants was reviewed and approved by the Centre for Addiction and Mental Health Research Ethics Board. Written informed consent to participate in this study was provided by the participants' legal guardian/next of kin.

## Author Contributions

NJK conceived and designed the study, was responsible for writing the first draft, and approved the final draft for submission. AS designed and conceived the study, was responsible for data collection, and approved the final draft for submission. GG was responsible for data collection and statistical analysis and approved the final draft for submission. KAK and JH was responsible for data collection and approved the final draft for submission. CH was responsible for statistical and MRI analysis and approved the final draft for submission. TAS, MW, and LA conceived and designed the study and approved the final draft for submission. All authors listed have made a substantial, direct, and intellectual contribution to the work and approved it for publication.

## Funding

This research was funded by an Ontario Mental Health Foundation New Investigator Fellowship and a Slaight Family Centre For Youth in Transition Research Award, both awarded to NJK.

## Conflict of Interest

The authors declare that the research was conducted in the absence of any commercial or financial relationships that could be construed as a potential conflict of interest.

## Publisher's Note

All claims expressed in this article are solely those of the authors and do not necessarily represent those of their affiliated organizations, or those of the publisher, the editors and the reviewers. Any product that may be evaluated in this article, or claim that may be made by its manufacturer, is not guaranteed or endorsed by the publisher.
